# Gross and Histopathologic Comparison of the Distal Third Metacarpal Bone and the Proximal First Phalanx with Sodium Fluoride Positron Emission Tomography Radiopharmaceutical Uptake in Five Horses

**DOI:** 10.3390/vetsci13060591

**Published:** 2026-06-18

**Authors:** Maureen Kelleher, Jacqueline Marr, Brittney Graham, Thomas Cecere, Brett Klamer, Sergey Anishchenko, David Beylin

**Affiliations:** 1 College of Veterinary Medicine, The Ohio State University, Columbus, OH 43210, USA; 2 College of Veterinary Medicine, University of Florida, Gainesville, FL 32611, USA; j.marr@ufl.edu; 3 Department of Anatomic Pathology, College of Veterinary Medicine, The Ohio State University, Columbus, OH 43210, USA; graham.700@osu.edu; 4 Virginia-Maryland College of Veterinary Medicine, Virginia Polytechnic Institute and State University, Blacksburg, VA 24061, USA; tcecere@vt.edu; 5 Center for Biostatistics, The Ohio State University, Columbus, OH 43210, USA; brett.klamer@osumc.edu; 6 Alienbyte Scientific Software Inc., Rockville, MD 20852, USA; 7 Division of Brain Biosciences, Inc., LONGMILE Veterinary Imaging, Rockville, MD 20852, USA; david@brain-bio.com

**Keywords:** equine, positron emission tomography, metacarpus, fetlock, phalanx

## Abstract

Injuries of the metacarpophalangeal joint are a major cause of morbidity and catastrophic fracture in racing horses, yet early osseous pathology is often difficult to detect using conventional imaging. This pilot study aimed to correlate sodium fluoride Positron Emission Tomography (^18^F-NaF PET) radiopharmaceutical uptake with gross and histopathologic changes in the distal third metacarpal bone (MC3) and proximal first phalanx (P1). Five horses (three racing Thoroughbreds with fetlock injury and two non-racing controls) underwent ante-mortem ^18^F-NaF PET and cone-beam CT imaging (CBCT), followed by post-mortem gross and histologic examination of predefined anatomic sites. Quantitative PET measures, including maximum standardized uptake value (SUV_max_), SUV_ratio_, and PET grade, were compared with gross pathology and histopathologic scores for cartilage and subchondral bone. While there were significant regional correlations between PET metrics and gross or histologic scores at select sites, our results need to be considered in light of the small number of horses evaluated. Correlations between PET metrics and gross pathology score were identified on the distal metacarpus on the lateral dorsal condyle and on proximal P1 for lateral dorsal and mid-P1. Correlation of PET metrics and hyaline cartilage histopathology scores were found for dorsal medial and lateral P1, parasagittal dorsolateral P1 and the medial parasagittal groove of MC3. Correlation of PET metrics and histologic subchondral bone scores were significant for medial palmar condyle, medial parasagittal groove, and parasagittal palmar lateral of MC3. For P1, PET metrics and histologic subchondral bone scores were significantly correlated for parasagittal mid-lateral and medial dorsal regions. Overall, these findings demonstrate that ^18^F-NaF PET identifies localized bone remodeling that corresponds to histologic and gross pathology at specific fetlock regions, supporting its utility for detecting osseous injury, although relationships varied by anatomic location. Further work with larger numbers of horses is needed.

## Simple Summary

Injuries of the fetlock joint are a common cause of career-ending injury in racing horses, yet early damage within the bone is often difficult to detect. This pilot study examined whether imaging which detects active bone remodeling, Positron Emission Tomography (PET), can be linked to the physical and microscopic damage seen in bone tissue. The study evaluated the distal metacarpus and proximal phalanx from five horses, including injured racehorses and control horses. Imaging findings were compared with visible damage to the joint surface and with changes seen histologically, after the horses were humanely euthanized. Findings suggest that PET can help identify areas of abnormal bone remodeling. Areas of higher activity on PET showed visible signs of stress-related damage and remodeling at a gross and histologic level, particularly in locations known to be vulnerable in racing horses. However, the relationships were not uniform across all regions.

## 1. Introduction

Metacarpophalangeal (MCP; fetlock) joint disease contributes significantly to racehorse morbidity and mortality [1,2,3,4,5]. Numerous studies have shown the importance of fetlock injuries on both racehorse and jockey welfare [3,4,5].

The stress and strain of exercise are known to traumatize bone and induce bone remodeling to adapt it to these forces. However, constant repetitive loading on the bone can result in damage accumulating faster than it can be remodeled to repair it. Inadequate bone remodeling leads to microscopic fractures and the potential for macrofracture [6]. Metacarpal condylar fractures occur in areas of bone that have been remodeled [7], have microdamage [3], and have structural change from fatigue [8].

Positron Emission Tomography (PET) uses 18-sodium fluoride (^18^NaF) as a marker of bone turnover, binding to exposed hydroxyapatite crystals. Recent studies using ^18^F-NaF PET in horses have reported excellent results for detecting and monitoring bone injury in the fetlocks, feet, and hocks [9,10,11,12]. Additionally, ^18^NaF-PET can monitor fetlock injury in racing horses to support appropriate return to training and racing, including after post-surgical repair [11,13,14]. A minimal description of the comparison between gross pathology and histopathology and PET radiopharmaceutical uptake has been reported [14].

Standardized uptake values (SUV) are quantitative measures of the radiopharmaceutical concentration at a specific region of interest. SUV maximum (SUV_max_) is the voxel in a region of interest with the highest radiopharmaceutical uptake (RU). In horses without subjectively increased radiopharmaceutical uptake, ^18^NaF SUV_max_ has been reported for the distal limb [15]. To understand another measure, SUV_ratio_, first the SUV_max_ in a universal target (clinically unaffected) region or “SUV_background(bg)_” is measured. SUV_ratio_ is a ratio of SUV_max_ at a specific anatomic site to SUV_bg_. Since each patient has different isotope uptake characteristics, SUV_ratio_ could improve the comparison of PET RU at specific anatomical sites for clinical re-evaluations and across equine PET studies [16]. Continued investigation of SUVs may lead to an acceptable range of normal objective PET values for specific anatomic locations [14]. Additionally, leading equine PET investigators have developed a grading scale for PET radiopharmaceutical uptake, based on the SUV_ratio_ where grade 1 (mild) is a calculated ratio of less than 2, grade 2 (moderate) is a calculated ratio between 2 and 3, and grade 3 (severe) is a calculated ratio greater than 3.

Given the sensitivity of PET for the detection of bone remodeling and the lack of correlation of gross and histopathology to PET radiopharmaceutical uptake, we aimed to correlate the gross and histologic characteristics of the distal metacarpal III (MC3) and proximal first phalanx (P1) with Positron Emission Tomography (PET) using three racing horses and two control horses. The goal of this investigation was to correlate standardized uptake values (SUV_max_, SUV_ratio_) and PET grade with gross pathologic and histologic scores in regions commonly associated with distal MC3 and proximal P1 bone disease in racing Thoroughbreds. We hypothesized that higher pathology and histopathology scores would correlate with higher SUV_max_, SUV_ratio_, and PET. However, given the timing of our investigation, the small sample size, and unfortunate processing errors, it should be viewed as a pilot study that yields valuable survey data to inform future studies.

In human medicine, metabolic tumor volume (MTV) quantifies metabolic activity within a selected area on PET imaging and is used prognostically to assess response to intervention. Compared with SUV_max_, which represents a single voxel in the selected region, metabolic volume calculations allow for background subtraction and threshold specification to remove clinically insignificant outliers, resulting in a better estimate of the metabolic burden of the entire tumor [17]. Metabolic volume has not been previously reported with equine PET imaging, but may better represent “injury volume” than SUV measures. An additional aim of this study was to measure MV at the same sites, coincident with the SUV measurements, and compare it with SUV_max_. For this secondary aim, we hypothesized that there would be no significant difference between SUVmax calculated from a single voxel and that calculated by MV.

## 2. Materials and Methods

Four Thoroughbred horses and 1 Warmblood horse were included in this study. Three horses, aged 4, 6, and 7, were actively racing until the point of injury. One Thoroughbred horse, aged 5, retired from racing for 16 months, was included as a control horse. One Warmblood horse, age 10, a former lower-level eventing horse, who was grade 3 lame and diagnosed with significant navicular degeneration via magnetic resonance imaging, was also included as a control case. The owners donated the horses for a terminal study. The institution’s International Animal Use and Care Committee approved the study (18-079). Each horse had a physical examination before anesthesia. After sterile preparation, a 14-gauge, 5.25″ intravenous catheter (Angiocath IV, Becton Dickinson, Franklin Lakes, NJ, USA) was placed in the left jugular vein and maintained until the time of death. Each horse was induced and maintained under general anesthesia until imaging was completed. Briefly, each horse was sedated to effect with xylazine (0.2–1.0 mg/kg) intravenously (IV) and induced with a combination of midazolam (0.1–1.0 mg/kg) and ketamine (2.2 mg/kg) IV. Horses were positioned in lateral recumbency with the affected limb upmost, and anesthesia was maintained with inhaled isoflurane (1.0–3.0%) in oxygen. If needed, horses were supplemented with dobutamine (1–5 µcg/kg/min) as a constant rate infusion to maintain mean arterial pressure. After all imaging, the horses were euthanized with sodium pentobarbital (1 mL/4.5 kg) via the intravenous catheter while under general anesthesia. After the appropriate radiation wash-out period, the distal aspect of each front limb was removed and stored frozen.

### 2.1. Image Acquisition

Each forelimb fetlock was placed in the center of a cone-beam computed tomographic (CBCT) scanner (Pegaso, Epica, Landrum, SC, USA) and imaged using a scan time of 53 s with a kVp of 80, mA of 110, 12 ms, standard field of view (FOV), panel height 50 cm, and the soft tissue filter. Once CBCT images were obtained, the horse was withdrawn from the CT but remained within the shielded CT suite. Thirty millicuries of ^18^F-NaF were injected through the intravenous jugular vein catheter and allowed to distribute through the body for a 45-min period. Each MCP joint was placed in the center of a 22 cm gantry of a portable human brain PET scanner (LONGMILE Veterinary Imaging, Rockville, MD, USA). Radioactivity was collected for an average of 12 min per joint.

CBCT and PET images were uploaded to a web-based imaging software (Bru^TM^; Alienbyte, Rockville, MD, USA). The CBCT images were co-registered with PET images for anatomical localization. Using a cuboidal selection tool, a 0.04 mm^3^ was selected at 15 locations on the distal aspect of MC3 (Figure 1) and eight sites on the proximal aspect of P1 (Figure 2) for calculation of SUV_max_, and PET grade. SUV_bg_ was obtained in the trabecular bone region of either the mid-distal MC3 or the proximal-mid aspect of the proximal P1 diaphysis using a 0.1–0.11 cm^3^ area in a uniform region without disease (Figure 3). SUV_ratio_ was determined from SUV_max_ and SUV_bg_. Using the same cuboidal areas, and a threshold of 2.5, metabolic volume (MV), was calculated for each of the study and background sites (Figure 4). SUV_max_, SUV_ratio_, PET grade, and MV data were downloaded from the software to a Microsoft Excel spreadsheet (Microsoft, Redmond, WA, USA).

### 2.2. Gross Pathology Methods

Each MCP joint was opened, and the surfaces of distal MC3 and proximal P1 were photographed and graded using a modification of an established grading system [18] at the 23 locations described (Table 1). The total range possible for gross scoring of distal MC3 was 0–14. The total range possible for gross scoring of proximal P1 was 0–8. Grading was performed by a board-certified pathologist (TC).

### 2.3. Histopathology Methods

The specimens suffered significant damage during storage and transport, making slide preparation difficult, and leaving many locations without usable data. After gross examination, representative sections of each area of interest were decalcified and routinely processed for histopathology. Each histologic region was graded using a modification of a previously described scale [19] by a board-certified pathologist (JM). The total range possible for the hyaline cartilage (HC) score was 0–15, and the total range for subchondral bone scores was 0–21. Table 2A lists the grading scale for hyaline cartilage, and Table 2B lists the subchondral bone scale.

### 2.4. Statistical Analysis

Descriptive statistics were used to summarize gross pathology, histopathology, and imaging variables within and across limb type (affected, contralateral, control) and location (23 sites). Pearson’s correlation was used to measure the association between imaging variables (SUV_max_, SUV_ratio_, and PET grade) and pathology variables (gross and histologic) within each location and across limb types. The paired t-test was used to assess within-horse differences between the affected and contralateral limbs for gross pathology, histopathology, and imaging variables at each location. Negative binomial regression models were used to perform likelihood ratio tests for differences in histopathology outcomes between limb types within P1 and MC3 regions, and results are interpreted under the assumption that observations are independent and identically distributed. Statistical significance was assessed at an unadjusted alpha level of 0.05, and all analyses were performed using R version 4.5.2.

## 3. Results

### 3.1. Horses

All three injury cases sustained acute proximal sesamoid bone (PSB) fractures, including bilateral fractures in one horse. The Thoroughbred control horse had retired for 16 months due to severe superficial digital flexor (SDF) tendonitis sustained during racing and never reached pasture soundness. The Warmblood was euthanized due to significant navicular degeneration. Both front limbs were available for the three racing horses and the Warmblood. Only the left limb, with the SDF tendonitis, was available for the retired Thoroughbred, resulting in nine limbs (five left, four right). Three horses had four proximal sesamoid bone fractures (two fractures in one horse and one fracture each in two horses). Fracture configurations included one basilar, one abaxial, and two apical, occurring on the medial PSB. Due to a storage error, all the PSBs were lost during the study; therefore, this anatomy was not included.

### 3.2. Gross Pathology

The mean total gross score for all locations of the distal MC was 0.97 (SD 1.25; range 0–6, median 1). Mean total gross score for affected limbs was (1.53, 1.42 SD), contralateral limbs (0.80, 1.01 SD), and for control limbs was 0.58 (1.08 SD). The medial parasagittal groove (Figure 5) was the region with the highest total mean gross score for all limbs (2.56, 2.01 SD), affected (4.33, 1.53 SD), and contralateral limbs (3.0, 1.0 SD). The region with the highest mean total gross score for control limbs was the lateral parasagittal groove (2.0, 1.73 SD) (Figure 6).

The mean total gross score for all locations on proximal P1 was 0.57, 0.60 SD (range 0–2, median 1). None of the proximal P1 regions exceeded 1 for the mean total gross score for all limbs combined. The parasagittal dorsolateral and parasagittal dorsomedial sites had the highest mean total gross score, 1.33 (SD 0.58).

### 3.3. Histopathology

The mean metacarpal hyaline cartilage score for all samples was 2.70 (3.5 SD) (range 0–14). The mean MC hyaline cartilage score for control limbs was 2.30 (3.2 SD) (range 0–14), with the mean for affected limbs 2.80 (3.6 SD) (range 0–12) and the contralateral limbs 4.0 (4.1 SD) (range 0–12) slightly higher. The mean distal MC3 hyaline cartilage scores for control, affected, and contralateral limbs were not significantly different from each other (*p* = 0.5). The region with the highest mean total HC score across all limbs was the medial mid-condyle, with scores available for 5 of 9 limbs (7.8, 5.2 SD) (Figure 7).

The distal metacarpal mean total subchondral bone (SCB) score for all samples was 2.75 (3.44 SD) (range 0–13). The metacarpal total mean SCB score for control limbs was 1.80 (2.50 SD) (range 0–10), which was lower than the mean SCB scores of affected limbs, 3.38 (4.18 SD) (range 0–13), and of the contralateral limbs, 5.00 (3.82 SD) (range 0–10). The mean distal MC3 SCB scores for control, affected, and contralateral limbs were not significantly different (*p* = 0.14). In the regions with the highest mean total SCB scores for all limbs, with five of nine limbs having scores available, the medial mid condyle had the highest mean total SCB score (8.0, 3.94 SD) (Figure 8).

The mean proximal P1 hyaline cartilage score for all samples was 2.00 (3.22 SD) (range 0–13). The mean P1 hyaline cartilage score for control limbs was 1.94 (4.25 SD) (range 0–13), slightly higher than the mean P1 hyaline cartilage score for affected limbs (Figure 9), 1.75 (2.14 SD) (range 0–6) but lower than the mean for contralateral limbs 2.50 (2.14 SD) (range 0–7). The mean proximal P1 hyaline cartilage scores for control, affected, and contralateral limbs were not significantly different from each other (*p* = 0.9). The region with the highest mean total HC score for all limbs was medial mid P1 (4.3, 7.5 SD), with only three of nine limbs having scores. The parasagittal dorsomedial region had the highest mean total HC score (4.50, SD 3.54) for contralateral limbs.

**Figure 7:**
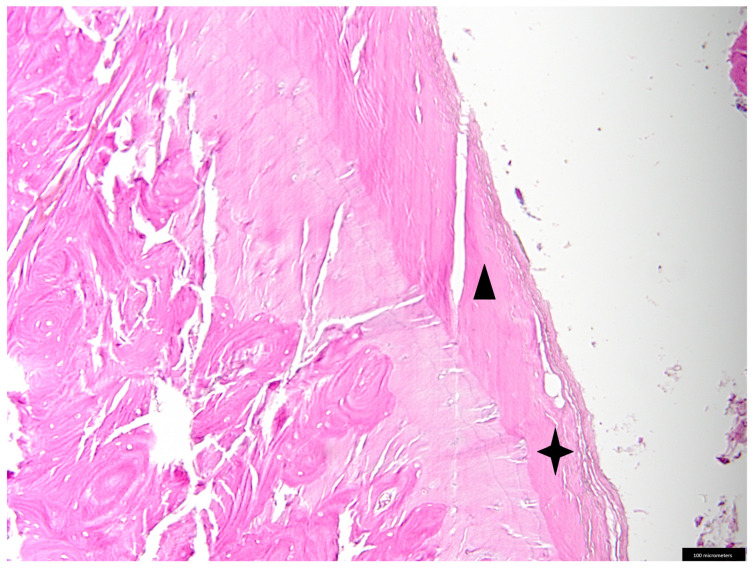
Representative degenerative histopathological findings of the right forelimb third metacarpal bone parasagittal groove hyaline cartilage featuring fibrillation (star) and marked chondrocyte dropout (triangle); 100×, H&E, (Scale bar = 100 μm). Photo: B. Graham.

**Figure 8:**
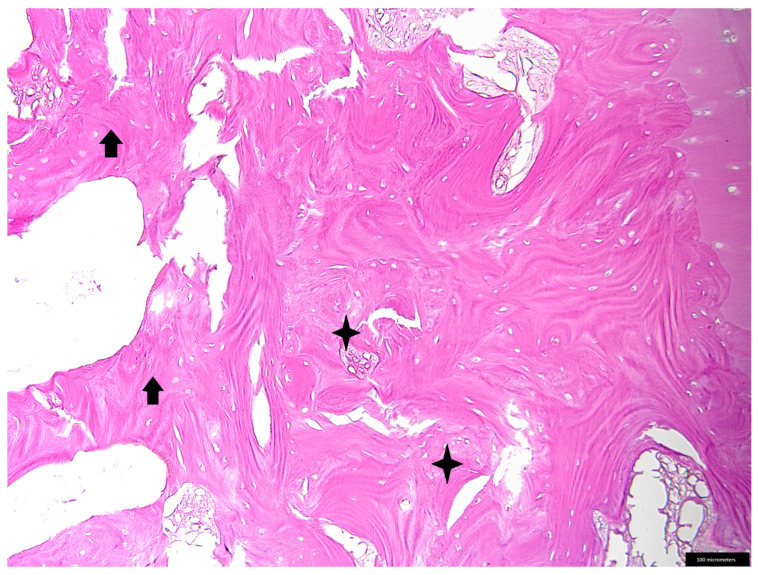
Representative degenerative histopathological findings of the left forelimb third metacarpal bone medial mid condyle subchondral bone featuring sclerosis of adjacent cancellous bone (arrows) with narrowing of marrow spaces and multiple regions of replacement of compact bone with woven bone (stars) and a lack of normal osteons (marked modeling); 100×, H&E, (Scale bar = 100 μm). Photo: B. Graham.

**Figure 9:**
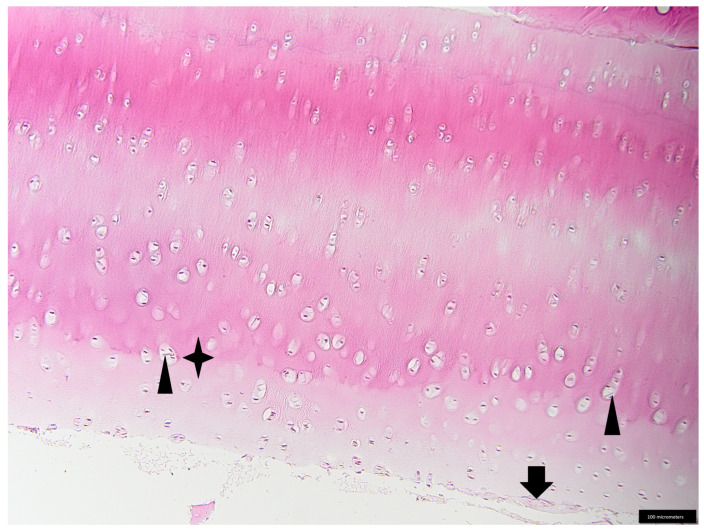
Representative degenerative histopathological findings of the left mid-lateral parasagittal hyaline cartilage of the first phalanx (P1) featuring irregularity of the articular surface with fibrillation (arrow), mild chondrocyte clustering (triangles) and irregular chondrocyte distribution (hypocellularity of the unmineralized cartilage) and regional thinning of the articular cartilage (indicative of ulceration; with the junction between mineralized and nonmineralized hyalin cartilage labeled with a star); 100×, H&E, (Scale bar = 100 μm). Photo: B. Graham.

For P1 subchondral scores, the mean for all samples was 4.2 (3.6 SD) (range 0–12). The mean P1 SCB score for control limbs, 5.10 (3.9 SD) (range 0–12), and contralateral limbs, 5.00 (3.5 SD), was higher than the mean score for affected limbs (Figure 10), 2.30 (2.7 SD). The mean proximal P1 SCB scores for control, affected, and contralateral limbs were not significantly different from each other (*p* = 0.17). The region with the highest mean total SCB scores for all limbs was medial mid-P1 (6.75, 4.72 SD), with four of nine limbs having scores and strongly influenced by the control limbs group, which had three of the four scores (9.0, 1.73 SD).

### 3.4. Imaging

The mean SUV_max_ across all locations on MC3 was 5.9 (SD 3.9). The mean SUV_max_ for the MC3 background (bg) was 2.80 (0.23 SD). For affected limbs, the mean SUV_max_ was 6.3 (3.9 SD), which was higher than the mean SUV_max_ of the control limbs (3.0, 0.4 SD) but lower than the contralateral limbs (7.1, 4.2 SD). The SUV_ratio_ across all locations on MC3 was 3.11 (SD 4.05). The SUV_ratio_ of affected limbs was 5.71 (6.09 SD), higher than both contralateral limbs (2.29, 1.34 SD) and control (1.33, 0.38 SD). The mean PET grade (0–3) for all limbs was 1.59 (0.93 SD) with the affected limb mean grade of 2.27 (0.81 SD), compared to 1.58 (0.89 SD) for contralateral limbs and 0.91 (0.51 SD) for control (Table 3). The region with the highest mean SUV_max_ (six of nine limbs)/SUV_ratio_ (nine of nine limbs)/PET grade (nine of nine limbs) for all limbs was the parasagittal palmar medial site.

The mean SUV_max_ across all locations on P1 was 5.2 (SD 3.9). For affected limbs, the SUV_max_ was 7.7 (6.1 SD), higher than the contralateral (5.4, 1.7 SD) and control (3.2, 0.5 SD) values. The SUV_ratio_ for all locations on P1 was 2.13 (1.37 SD), with the affected mean ratio of 2.64 (1.81 SD), contralateral 2.07 (1.31 SD), and control 1.67 (0.55 SD). The mean PET grade for P1 was 1.46 (SD 0.79). The mean PET grade for the affected limbs was 1.79 (SD 0.88), which was higher than both the contralateral (1.29, SD 0.86) and control (1.29, SD 0.46) limbs. For the lateral dorsal region of P1, there was a significant difference in mean SUV_max_ (*p* = 0.048) between affected limbs, 17.7 (0.9 SD), and contralateral limbs, 4.3 (0.5 SD). Additionally, for the lateral dorsal region of P1, there was a significant difference in mean PET grade (*p* = 0.038) between affected limbs, 2.33 (0.58 SD), and contralateral limbs, 0.67 (0.58 SD) (Table 4). The region with the highest mean SUV_max_/SUV_ratio_/PET grade for all and contralateral limbs was parasagittal dorsolateral. For affected limbs, the region was lateral dorsal, and for control limbs, it was medial mid P1.

A comparison of SUVmax calculated using the single voxel within the 0.4 mm^3^ regions and the metabolic volume with a threshold of 2.5 and background subtraction at all sites in all limbs was not significantly different.

### 3.5. Correlations

Correlation between SUV_max_ and total gross score was 0.99 (95% CI: 0.95, 1; n = 6) for lateral dorsal P1 and 0.95 (95% CI: 0.71, 0.99; n = 7) for lateral dorsal condyle of MC3. Correlation between SUV_max_ and total HC score was 0.98 (95% CI: 0.5, 1; n = 4) for dorsomedial P1 and 0.97 (95% CI: 0.6, 1; n = 5) for dorsolateral P1. Correlation between SUV_max_ and total SCB score was 0.95 (95% CI: 0.39, 1; n = 5) for parasagittal palmar lateral MC3 (Table 5).

Correlation between SUV_ratio_ and total gross score was 0.78 (95% CI: 0.24, 0.95; n = 9) for lateral mid-P1 and 0.69 (95% CI: 0.05, 0.93; n = 9) for lateral dorsal P1. Correlation between SUV_ratio_ and total histologic hyaline cartilage score was 0.99 (95% CI: 0.85, 1; n = 4) for dorsal medial P1 and 0.95 (95% CI: 0.4, 0.99; n = 5) for parasagittal dorsolateral P1. Correlation between SUV_ratio_ and total histologic subchondral bone score was 0.99 (95% CI: 0.91, 1; n = 4) for dorsal medial P1, 0.97 (95% CI: 0.65, 1; n = 5) for medial palmar MC3 condyle, 0.95 (95% CI: 0.4, 1; n = 5) for parasagittal groove medial MC3, and −0.83 (95% CI: −0.98, −0.05; n = 6) for parasagittal mid-lateral P1 (Table 6).

Correlation between PET grade and total gross score for lateral dorsal P1 was 0.76 (95% CI: 0.18, 0.95). Correlation between PET grade and total HC score was 0.99 (95% CI: 0.87, 1; n = 4) for dorsal medial P1 and 0.88 (95% CI: −0.03, 0.99; n = 5) for the medial parasagittal groove of MC3. Correlation between PET grade and total SCB score was 0.84 (95% CI: −0.17, 0.99; n = 5) for medial palmar MC3 condyle (Table 7).

## 4. Discussion

The original aim of this project was to correlate measures of PET uptake (SUV_max_, SUV_ratio_, and PET grade) with gross pathologic and histologic scores in regions commonly associated with metacarpophalangeal joint injury in racing horses. However, due to the timing of the study, we recruited only five horses before the data collection ceased. Subsequently, after dissection, a storage error occurred, resulting in loss of the proximal sesamoid bones (PSB), which is a significant contributor to MCPJ disease in racing Thoroughbreds [1,2] and the cause of donation-euthanasia for the three racing horses. While PET data on the PSB was collected, there was incomplete gross pathologic data and no histopathologic data. The loss of the PSB is a significant limitation on the original aim of this project. Lastly, a freezer issue damaged the bones, resulting in incomplete data sets across many sites and further degrading the quality of the information. Despite the numerous adversities the project faced, we aimed to determine whether higher pathology and histopathology scores would correlate with higher SUVmax, SUVratio, and PET grade, with the understanding that the small number of horses and limbs would be unlikely to yield statistically significant results, yet could still be descriptively beneficial to the report.

In racing Thoroughbreds, predilection sites of bone injury at the distal aspect of MC3 are the medial and lateral palmar parasagittal groove and the mid sagittal groove, which have been noted grossly [20] and histologically [21]. These sites of subchondral bone remodeling and fatigue increase the risk of condylar fracture [3,7,8]. Grossly, the medial parasagittal groove was the region with the highest scores across all affected and contralateral limbs, whereas for control limbs, the lateral parasagittal groove was highest-scoring, consistent with previous reports [20,21]. Histologically, our results differed slightly, with the MC3 medial mid condyle being the most affected region for hyaline cartilage changes (for all affected and contralateral limbs). Similarly, the MC3 medial midcondyle was the highest scoring for all affected and contralateral limbs for subchondral bone changes. For affected limbs, the medial parasagittal groove was equally scored with the medial mid condyle for hyaline cartilage. As mentioned previously, the differences between our study and other published studies are likely due to sample size and the loss of various frozen samples for histopathology.

When subchondral bone scores were correlated with PET grading, SUV_ratio_ and SUV_max_, there were significant correlations between imaging and histologic values in areas of bony remodeling related to prodromal condylar fracture, mainly along medial and lateral parasagittal regions, which are at increased condylar fracture risk [3,7,8,22]. While the number of horses is low, three horses with PSB fracture each had significant gross articular cartilage pathology and histopathologic hyaline cartilage and subchondral bone disease of MC3. These horses did not suffer condyle fractures, but our results suggest that PET may help evaluate the risk of PSB fractures earlier (when there is only microscopic evidence of articular disease) compared with other imaging modalities.

Radiography has been used as a first-line modality for fetlock imaging for decades, but primarily as a diagnostic tool for established clinical disease, rather than a screening tool for the accumulation of subchondral bone injury prior to clinical signs/architectural failure/fracture. Studies have shown radiography to be insensitive in the assessment of subchondral bone sclerosis [23,24,25] despite its significant association with microscopic damage accumulation and risk of condylar fracture [3,4,7]. In recent years, computed tomography (CT) has been found to be superior to radiography for the detection of subchondral bone lysis [23,24,25], both associated with condylar fracture [22]. Other studies have compared CT with magnetic resonance imaging (MRI) for detection of bone remodeling associated with metacarpal condylar fracture and articular cartilage injury [23,24,25,26,27] with both modalities having advantages and disadvantages. Low-field MRI lacks the ability to detect articular cartilage injury but can be performed while standing, with the added benefit of allowing severity assessment based on signal intensity changes in specific MRI sequences [27]. The availability of standing MRI and CT is broadening in the racing industry, with some jurisdictions, such as Racing Victoria in Australia, utilizing standing CT scans for screening of horses before highly publicized races [25]. However, while CT provides sensitive and specific structural data [25], no functional bone tissue data is provided. MRI interpretation, while it provides modest functional information, often underestimates disease of the metacarpal condyles [24,27]. PET, on the other hand, provides no structural data but offers excellent functional data on stress remodeling in the racehorse fetlock joint and can be used alone or in combination with CT or MRI to add pertinent structural information. The utility of monitoring racehorse MCP joints for the risk of catastrophic breakdown and to determine an appropriate lay-up has been suggested previously [11] as has recurrent monitoring for post-operative condylar fracture repair for complications, such as the development of osteoarthritis [13]. Each imaging modality, used in isolation, has been reported to have deficits in detecting diseases that pose a health risk to the horse and jockey. Despite these deficits, intensity scores for bony remodeling recognized with PET may have significant utility in reducing the incidence of breakdown injuries, especially when paired with a standing modality that provides superior structural information, such as CT. From a cost-risk perspective, PET, with or without complementary CT, carries a low risk of injury during acquisition because imaging is performed while standing and, anecdotally, appears affordable to many racehorse owners (personal communication).

There is a smaller body of literature reporting the effects of racing on proximal P1 injury compared with the distal aspect of MC3. Two recent studies [21,28] reported histological and three-dimensional imaging features of the sagittal groove of proximal P1, indicating that the sagittal groove has considerable accumulation of stress remodeling. Additionally, an increase in subchondral bone sclerosis is seen in 2-year-olds in training, most significantly in the medial and lateral parasagittal ridges of P1. However, measurements were acquired in a single plane, specifically the frontal plane, through mid-P1 [29]. Similarly, prior to this work, Noble et al. [30] found that the subchondral bone thickness of proximal P1 was greater centrally and thicker dorsally when both were compared to the palmar subchondral bone. In the current study, the highest mean scores for gross, histopathology, and PET scores were either in the mid- and dorsal portions of P1. PET measures correlate with both HC and SBC values in these regions, suggesting that PET is sensitive for determining bone remodeling in these specific regions of stress [28]. Our study did not investigate sites on the palmar aspect of P1, so sensitivity for detecting microscopic remodeling at this site is unknown. Additionally, the published literature investigating the influence of P1 stress and remodeling on racehorse catastrophic breakdown injuries emphasizes the importance of trauma to the sagittal groove [21,28], but our study did not collect data from this region of the bone.

Our study results loosely suggest that higher gross pathology scores of the lateral dorsal condyle of MC3 correlate with higher SUV_max_, but A A larger sample of horses is indicated to test the hypothesis that higher gross and histologic pathology scores are correlated with PET scores. However, this location, the lateral dorsal condyle, has not been reported as a prioritized area of interest in relation to propagation of condylar fractures or development of palmar osteochondral disease. Similarly, for P1, the gross-PET score was only statistically significant for one of eight regions, the lateral dorsal aspect, but a large group of horses is needed to test the hypothesis. This may only be relevant to the precipitation of osteochondral fragmentation of proximal P1, but dorsomedial fragmentation is more common in forelimbs than lateral [31].

Our hypothesis that regions with higher histopathology scores would correlate with higher SUVmax was partially supported, but, as previously mentioned, but thehypothesis should be tested it in the future with a greater number of horses. For hyaline cartilage, SUV_max_, SUV_ratio_, and PET grade were not significantly correlated with any of the fifteen sites in distal MC3, except for the medial parasagittal groove with PET grade (Figure 11 and Figure 12). There were significant correlations between SUV_max_, SUV_ratio_, and PET grade and hyaline cartilage scores for proximal P1, including dorsal medial and lateral, and parasagittal dorsolateral.

With regard to subchondral bone, SUV_max_, SUV_ratio_, or PET grade were significantly correlated with SCB grade at the medial palmar condyle, a common site of palmar osteochondral disease, and the medial parasagittal groove (Figure 12), a location for condylar fracture propagation [3,7,8,22], and the parasagittal palmar lateral condyle. The PET measures were also significantly correlated at two P1 locations, including dorsal medial P1 and the parasagittal mid-lateral subchondral bone.

As noted throughout, the study had many limitations, including small sample sizes of horses and limbs, exclusion of hind limbs, loss of sesamoid bones, and specimen damage, which made histopathologic scoring challenging. Additionally, in retrospect, the inclusion of the sagittal groove and the palmar aspect of P1 would be valuable. Furthermore, unknown to the investigators at the outset of the study, the Warmblood control horse had marked fetlock pathology in one fetlock, skewing the control data, compared to the affected and contralateral limbs (Figure 13).

## 5. Conclusions

Despite numerous limitations, this study is a step toward understanding the link between sodium fluoride bone uptake, imaged with Positron Emission Tomography, and cartilage and subchondral bone histopathology, which could lead to metacarpophalangeal bone disease, including fracture. The only other literature on the histopathology of MC3 and PET is from one horse [14]. Our small project is a valuable addition to the body of literature relating microscopic subchondral bone and cartilaginous disease with PET imaging findings, benefiting the worldwide horse racing community as well as other equestrian sport stakeholders. Our exploratory project suggests that increased PET radiopharmaceutical uptake could serve as a beneficial imaging indicator of excessive or abnormal bone remodeling. At known predilection fracture sites, this data could be incorporated as a screening tool to inform changes in training, regardless of other imaging modalities, such as computed tomography or radiography, which can also be performed in the unanesthetized horse. Further investigation with larger groups of racing and non-racing horses, with and without fetlock-region pathology, is warranted to explore the utility of PET as a valuable screening tool for predicting catastrophic breakdown injuries and to inform training practices that promote horse welfare. Future studies should include evaluation of MC3, P1, proximal sesamoid bones, and likely an expansion of histopathology stains.

## Figures and Tables

**Figure 1:**
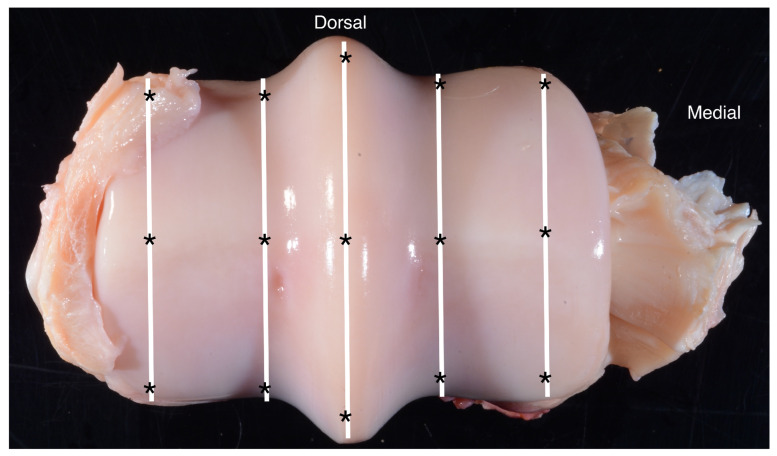
Gross image of the articular surface of a right front distal metacarpus showing the five planes and fifteen sites of investigation (asterisk). Photo: T. Cecere.

**Figure 2:**
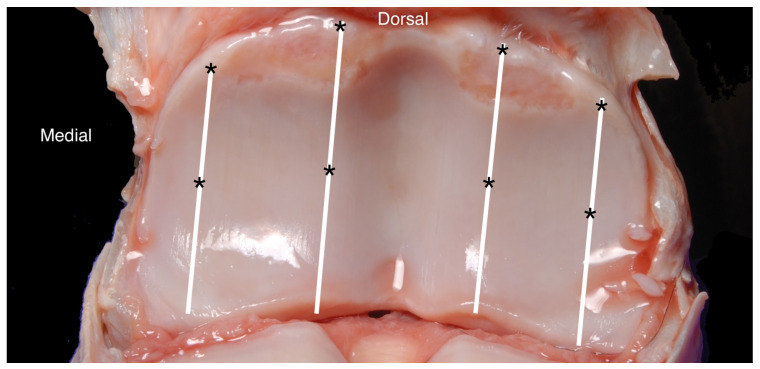
Gross image of the articular surface of a right front proximal first phalanx showing the four planes and eight sites of investigation (asterisk). Photo: T. Cecere.

**Figure 3:**
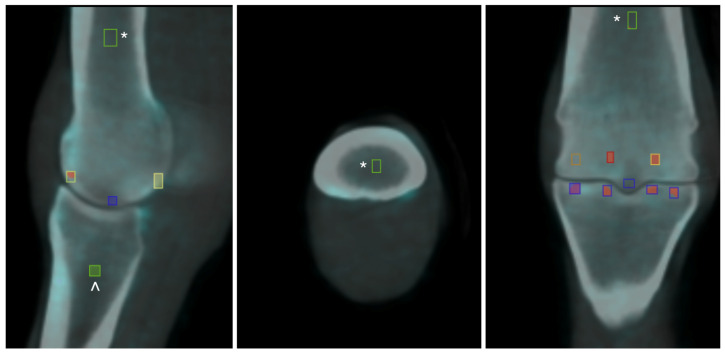
The SUV_max_ in the trabecular bone region free of disease the mid-distal MC3 (asterisk) and the proximal-mid aspect of the proximal P1 (arrowhead) was calculated from a 0.1–0.11 cm^3^ area and used for SUV_bg_. Image created using Alienbyte Scientific Software.

**Figure 4:**
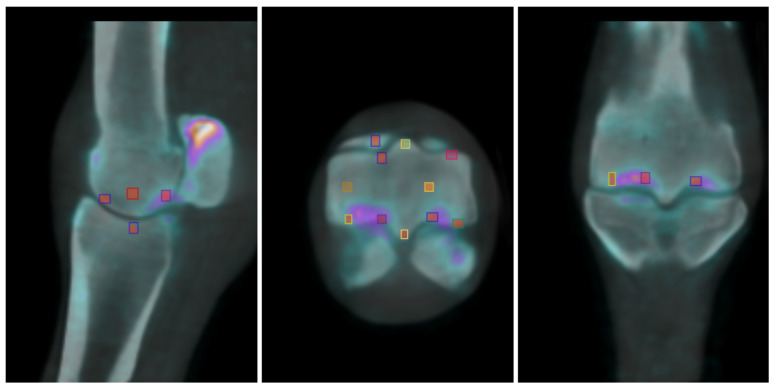
Cross-sectional image showing multiple 0.04 mm^3^ locations on the distal metacarpus measured to obtain various SUV measurements, metabolic volume (MV), and the PET grade at the fifteen sites. Image created using Alienbyte Scientific Software.

**Figure 5:**
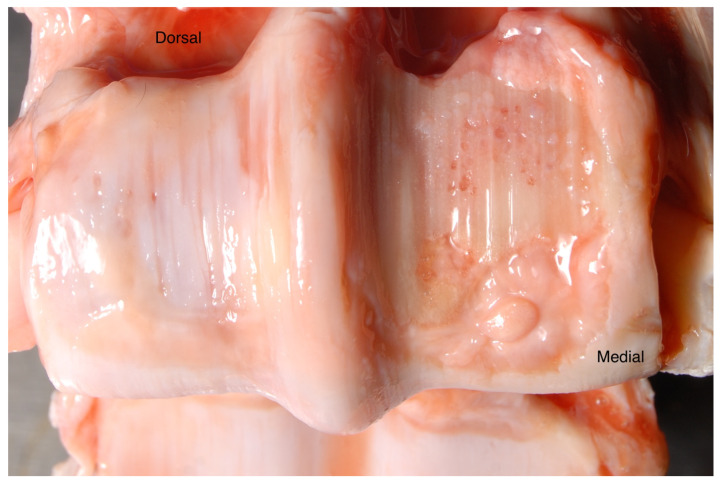
Left front distal metacarpus with a total score of six (maximum of 14) on the gross scoring of the medial parasagittal groove. This region had the highest total mean score for all limbs, affected limbs, and contralateral limbs. Photo: T. Cecere.

**Figure 6:**
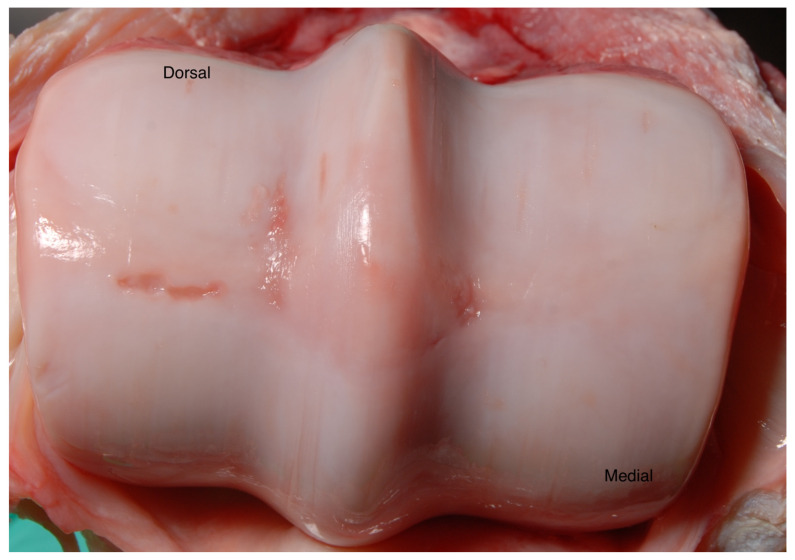
Left front metacarpus from the control Thoroughbred gelding retired from racing showing a cumulative grade 4 score out of a maximum of 14 at the lateral parasagittal groove, which was the region with the highest total mean score for control limbs. Photo: T. Cecere.

**Figure 10:**
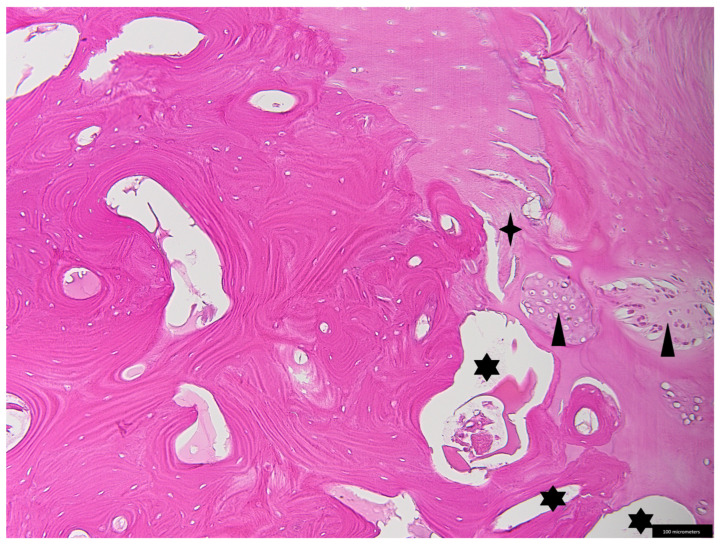
Representative degenerative histopathological findings of the right forelimb parasagittal dorsomedial P1 subchondral bone featuring increased porosity of compact subchondral bone indicative of disuse osteopenia (labeled with 6-pointed star; resorption without replacement suggestive of lack of mechanical use); 100×, H&E, (Scale bar = 100 μm). Adjacent chondrone formation (triangles), marked hypocellularity with proteoglycan loss, and fissuring of the mineralized hyalin cartilage (four-pointed star) are also apparent. Photo: B. Graham.

**Figure 11:**
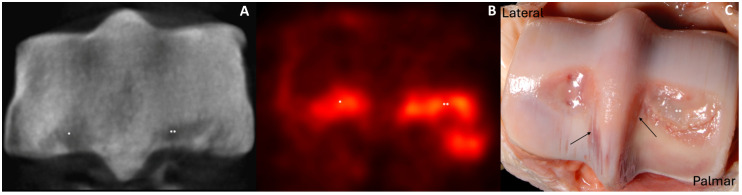
From left to right, (**A**) Computed Tomographic, (**B**) Positron Emission Tomographic, and (**C**) gross anatomic transverse images of the right front distal metacarpus of a 5-year-old Thoroughbred mare, donated to the study for bilateral medial proximal sesamoid fractures. Note there are significant wear lines noted on the gross image in both the medial and lateral parasagittal grooves (black arrows). Additionally, note there is significant increased radiopharmaceutical uptake of the lateral (one star) and medial condyles (two stars) that is associated with significant cartilage subchondral bone degeneration grossly and bone lysis on computed tomography.

**Figure 12:**
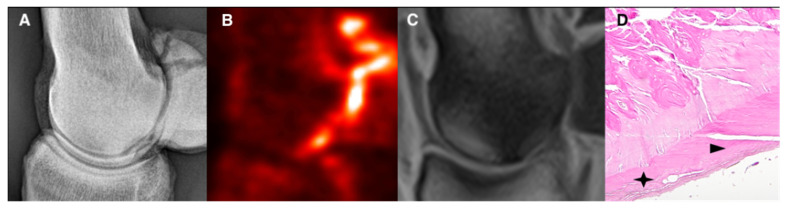
From left to right, (**A**) lateral radiograph, (**B**) sagittal Positron Emission Tomographic (PET) image, (**C**) sagittal T1-GRE magnetic resonance image (MRI), and (**D**) histopathologic image of the same mare in Figure 11. There is marked chondrocyte drop out (black triangle, right most image) in regions of increased signal intensity on MRI, increase radiopharmaceutical uptake on PET, and radiographic lucency.

**Figure 13:**
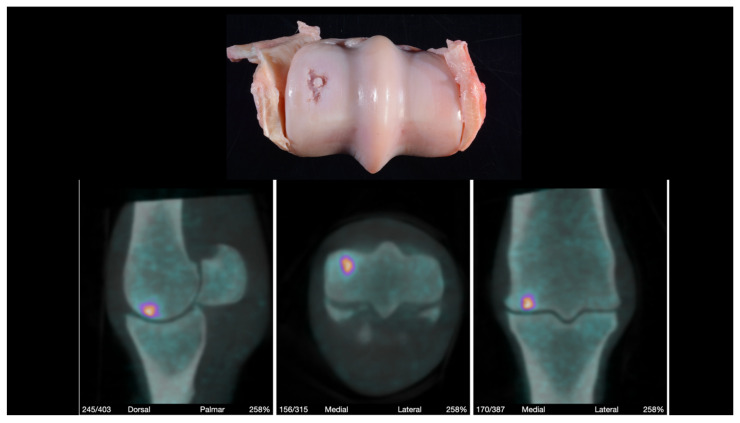
Left front fetlock of a Warmblood mare used as a control case in the study, euthanized for significant navicular degeneration diagnosed by diagnostic anesthesia, magnetic resonance imaging, and Positron Emission Tomography. The dorsal and mid-medial regions of the distal metacarpus had a significant, previously unknown cartilage and subchondral bone lesion, which was also evident histologically. On the fused PET-Computed Tomographic image, there is intense isotope uptake, shown as the focal region of intense yellow with a purple rim.

**Table 1:** Modification of established grading system [18] used to score the gross articular surfaces of MC3 and P1.

Feature	Score	Description
Palmar osteochondral disease (MC3 only)	0	No evidence of disease
	1	Discoloration of subchondral bone only, no or minimal disruption of overlying articular cartilage
	2	Discoloration with mild to moderate disruption of articular cartilage
	3	Established POD lesion. Discoloration and disruption of articular cartilage
Wear lines in cartilage	0	Wear lines absent
	1	Partial thickness wear lines in cartilage
	2	Full thickness wear lines in cartilage
Cartilage loss (transverse ridge, condylar groove, central condylar region—MC only)	0	No evidence of cartilage loss
	1	Partial thickness cartilage loss (fibrillation)
	2	Full thickness cartilage loss (ulceration) with minor exposure of subchondral bone
	3	Extensive full thickness cartilage loss with exposure of >5 mm diameter subchondral bone
Linear fissures	0	No evidence of linear fissures
	1	Faint groove with intact cartilage visible along length
	2	Well defined groove with partial thickness split in cartilage
	3	Well define groove with full thickness split in cartilage
Marginal remodeling	0	Absent
	1	Present
Dorsal impact injury	0	No evidence of impact injury
	1	Mild thickening of synovial pad and erosion of underlying MC3; mild erosion of dorsoproximal P1
	2	Severe thickening of the synovial pad, marked erosion of MC3; full cartilage loss or osteochondral fragment of proximal P1

**Table 2:** (**A**) Histologic grading of hyaline cartilage. Modification of established grading system [19] used to score the surfaces of MC3 and P1. (**B**) Histologic grading of subchondral bone. Modification of established grading system [19] used to score the surfaces of MC3 and P1.

**(A)**	
**Feature**	**Grading**
Sclerosis of the subchondral plate and adjacent cancellous bone	0 = No sclerosis, 1 = Mild, focal, 2 = Moderate, focal to more extensive sclerosis, 3 = Severe sclerosis, extending into non-weight bearing areas
Areas of subchondral bone collapse	0 = No collapse, 1 = Small, discrete area of collapse, 2 = Focal necrosis of the subchondral plate and minor hemorrhage, 3 = Collapse of the subchondral plate with hemorrhage/hematoidin
Obliteration of cancellous areas with compact bone	0 = Expected width of cancellous area, 1 = Focal and minimal, 2 = Moderate, 3 = Marked and/or in areas that bear no weight
Replacement with woven bone	0 = No lamellar bone deposit, 1 = Occasional discrete areas, 2 = Moderate, focal to more extensive replacement, 3 = Marked replacement
Replacement with osteonal bone	0 = No lamellar bone deposit, 1 = Minimal, 2 = Moderate, focal to more extensive, 3 = Marked deposition of lamellar bone
Increase in trabecular width with reduction of marrow spaces	0 = No thickening of trabeculae, 1 = Focal and minimal, 2 = Moderate thickening, 3 = Marked thickening and thickening in areas that bear no weight
Presence of Howship’s lacunae with and without osteoclasts	0 = No lacunae, 1 = Discrete and few, 2 = Multifocal, 3 = Numerous resorption lacunae
**(B)**	
**Feature**	**Grading**
Regularity of articular surface	0 = Normal, 1 = Mild irregularity, 2 = Moderate irregularity, 3 = Severe irregularity
Fibrillation	0 = No fibrillation, 1 = Mild fibrillation, 2 = Moderate fibrillation, 3 = Severe fibrillation
Articular cartilage thickness variation	0 = Expected thickness for the area, 1 = Mild variation, 2 = Moderate variation, 3 = Severe, extensive, or focal variation
Chondrocyte clustering	0 = Normal appearance of chondrocytes and lacunae, 1 = Formation of double chondrocytes, 2 = Presence of triplet chondrocytes and loss of linearity, 3 = Large numbers of chondrocytes clustered together within single supersized lacunae
Irregular distribution of chondrocytes	0 = Orderly distribution of chondrocytes, 1 = Focal areas with fewer or increased numbers of chondrocytes than expected, 2 = Focal areas with moderate variations in numbers, 3 = Extensive and severe alterations in chondrocyte distribution

**Table 3:** Descriptive table stratified by site and limb type (averages across label names, excluding background).

Characteristic	Affected	Contralateral	Control	Overall
MC3, n = 135 (total site measured)				
SUV_ratio_				
Number of sites	45	45	45	135
Mean (SD)	5.71 (6.09)	2.29 (1.34)	1.33 (0.38)	3.11 (4.05)
Median (Min, Max)	2.84 (1.14, 36.50)	1.98 (0.80, 7.57)	1.27 (0.72, 2.29)	1.84 (0.72, 36.50)
PET Grade				
Number of sites	45	45	45	135
Mean (SD)	2.27 (0.81)	1.58 (0.89)	0.91 (0.51)	1.59 (0.93)
Median (Min, Max)	2 (1, 3)	1 (0, 3)	1 (0, 2)	1 (0, 3)
SUV_max_				
Number of sites	39	30	18	87
Mean (SD)	6.3 (3.9)	7.1 (4.2)	3.0 (0.4)	5.9 (3.9)
Median (Min, Max)	4.7 (2.7, 17.6)	5.2 (3.0, 19.0)	3.0 (2.6, 3.8)	4.6 (2.6, 19.0)

**Table 4:** Descriptive table stratified by site and limb type (averages across label names, excluding background).

Characteristic	Affected	Contralateral	Control	Overall
P1, n = 72				
Ratio				
Number of sites	24	24	24	72
Mean (SD)	2.64 (1.81)	2.07 (1.31)	1.67 (0.55)	2.13 (1.37)
Median (Min, Max)	2.10 (1.16, 8.48)	1.74 (0.64, 7.18)	1.35 (1.01, 2.84)	1.81 (0.64, 8.48)
Grade				
Number of sites	24	24	24	72
Mean (SD)	1.79 (0.88)	1.29 (0.86)	1.29 (0.46)	1.46 (0.79)
Median (Min, Max)	1.5 (1, 3)	1.0 (0, 3)	1.0 (1, 2)	1.0 (0, 3)
SUVmax				
Number of sites	16	16	21	53
Mean (SD)	7.7 (6.1)	5.4 (1.7)	3.2 (0.5)	5.2 (3.9)
Median (Min, Max)	5.0 (2.7, 21.9)	4.7 (3.8, 9.3)	3.0 (2.7, 4.7)	4.0 (2.7, 21.9)

**Table 5:** Correlation and number of measures included for SUV_max_ and total gross, hyaline cartilage, and subchondral bone.

Score	Site	Correlation (95% Confidence Interval)	Number of Measures
Total gross score	Lateral dorsal condyle (MC3)	0.95 (0.71, 0.99)	7
	Lateral dorsal (P1)	0.99 (0.95, 1)	6
Hyaline cartilage score	Dorsomedial (P1)	0.98 (0.5, 1)	4
	Dorsolateral (P1)	0.97 (0.6, 1)	5
Subchondral bone score	Parasagittal palmar lateral (MC3)	0.95 (0.39, 1)	5

**Table 6:** Correlation and number of measures included for SUV_ratio_ and total gross, hyaline cartilage, and subchondral bone.

Score	Site	Correlation (95% Confidence Interval)	Number of Measures
Total gross score	Lateral mid (P1)	0.78 (0.24, 0.95)	9
	Lateral dorsal (P1)	0.69 (0.05, 0.93)	9
Hyaline cartilage score	Dorsomedial (P1)	0.99 (0.85, 1)	4
	Parasagittal dorsolateral (P1)	0.95 (0.4, 0.99)	5
Subchondral bone score	Medial palmar condyle (MC3)	0.97 (0.65, 1)	5
	Medial parasagittal groove (MC3)	0.95 (0.4, 1)	5
	Dorsal medial (P1)	0.99 (0.91, 1)	4
	Mid-lateral parasagittal (P1)	−0.83 (−0.98, −0.05)	5

**Table 7:** Correlation, 95% confidence interval, and number of measures included for PET grade and total gross, hyaline cartilage, and subchondral bone.

Score	Site	Correlation (95% Confidence Interval)	Number of Measures
Total gross score	Lateral dorsal (P1)	0.76 (0.18, 0.95)	9
Hyaline cartilage score	Medial parasagittal groove (MC3)	0.88 (−0.03, 0.99)	5
	Dorsal medial (P1)	0.99 (0.87, 1)	4
Subchondral bone score	Medial palmar condyle (MC3)	0.84 (−0.17, 0.99)	5

## Data Availability

The data presented in this study are available on request from the corresponding author due to privacy restrictions.

## Abbreviations

The following abbreviations are used in this manuscript:

MRI	Magnetic Resonance Imaging
PET	Positron Emission Tomography
SUV	Standardized Uptake Value
IRU	Increased Radiopharmaceutical Uptake
MC3	Metacarpal Three
P1	First Phalanx
F-NaF	Sodium Fluoride
MCP	Metacarpophalangeal (joint)
RU	Radiopharmaceutical Uptake
CBCT	Cone-Beam Computed Tomography
MC	Metacarpus
FOV	Field of View
HC	Hyaline Cartilage
SCB	Subchondral Bone
SD	Standard Deviation
